# Analysis of Additional Degrees in Academic Plastic Surgery Faculty

**DOI:** 10.1177/22925503221144039

**Published:** 2022-12-19

**Authors:** Sahil Chawla, Sarim Faheem, Michael L. Moreton, Amardeep Sekhon, Orapin M. Amornteerasawas, Jeffrey Ding, Faisal Khosa

**Affiliations:** 1 Faculty of Medicine, 8166 University of British Columbia, Vancouver, BC, Canada; 2 Faculty of Science, 8166 University of British Columbia, Kelowna, BC, Canada; 3 Faculty of Science, 8166 University of British Columbia, Vancouver, BC, Canada; 4 Department of Radiology, 8167 Vancouver General Hospital, Vancouver, BC, Canada

**Keywords:** academic, plastic surgery, faculty, degrees, advanced degree, chirurgie plastique, diplômes, diplôme avancé, professeurs, universitaire

## Abstract

**Background:** As plastic surgery continues to evolve, an increasing number of surgeons are attaining additional degrees (ADs). Prior studies illustrate this trend of increased AD attainment among plastic surgery faculty within the United States. Yet, no such study has documented AD attainment variability and influence within Canadian plastic surgery faculty. **Objectives:** Our objective was to investigate the relationship between AD attainment and gender, alongside research productivity, and academic rank of Canadian plastic surgery faculty members. **Methods:** All Canadian academic plastic surgery faculty members were identified and information regarding gender, academic rank, research productivity, timing of AD attainment was recorded. AD was defined as any degree beyond a medical degree or equivalent. **Results:** A total of 299 faculty members were identified. Of these, 33% (N = 99) attained an AD. A higher percentage of females (40%) obtained ADs compared to males (30%) (*P* = .0402). When controlling for number of years in practice, there was a significantly larger proportion of females than males with ADs as assistant and associate professor (*P* = .033). Faculty with ADs were associated with higher research productivity and higher academic rank than those with MDs (*P* < .05). ADs were commonly obtained post-residency (38%) and most common ADs were MSc (51%) and PhDs (21%). It was found that the Canadian plastic surgeons were less likely to pursue MBAs than US plastic surgeons (*P* = .002). **Conclusion:** One-third of Canadian academic plastic surgeons had ADs. Those with ADs present with higher research productivity and academic rank. When segmented by gender, there were significant differences among AD holders. The results of this study will lend support to ongoing endeavors voicing the need for gender equity in academic plastic surgery.

## Résumé

**Historique :** Tandis que la chirurgie plastique continue d’évoluer, de plus en plus de chirurgiens obtiennent des diplômes supplémentaires (DS). Des études antérieures démontrent cette tendance des professeurs en chirurgie plastique des États-Unis à obtenir des DS. Pourtant aucune étude n’a évalué la variabilité et l’influence des DS chez les professeurs de chirurgie plastique au Canada. **Objectifs :** Les chercheurs avaient comme objectif d’explorer la relation entre l’obtention de DS et le genre, conjointement avec la productivité de la recherche et le rang universitaire des professeurs de chirurgie plastique canadiens. **Méthodologie :** Les chercheurs ont répertorié tous les professeurs de chirurgie plastique canadiens et ont consigné l’information relative à leur genre, à leur rang universitaire, à la productivité de leur recherche, au moment de l’obtention des DS. Le DS était défini comme tout diplôme obtenu après un diplôme en médecin ou un diplôme équivalent. **Résultats :** Au total, 299 professeurs ont été répertoriés. De ce nombre, 33 % (N = 99) ont obtenu un DS. Un plus fort pourcentage de femmes (40 %) que d’hommes (30 %) (*P* = ,0402) a obtenu des DS. Après avoir tenu compte du nombre d’années d’exercice, une proportion beaucoup plus importante de femmes que d’hommes ayant des DS étaient professeurs adjoints et agrégés (*P* = 0,033). Les professeurs qui possèdent des DS étaient associés à une plus grande productivité de la recherche et à un rang universitaire plus élevé que ceux qui possédaient un diplôme en médecine (*P* < 0,05). Les DS étaient souvent obtenus après la résidence (38 %), et les plus courants étaient des maîtrises (51 %) et des doctorats (21 %). Il a été établi que les chirurgiens plasticiens canadiens étaient moins susceptibles de faire un MBA que les chirurgiens des États-Unis (*P* =0 ,002). **Conclusion :** Le tiers des chirurgiens plasticiens universitaires canadiens possédaient des DS. Ceux qui en détenaient présentaient une plus grande productivité en recherche et un meilleur rang universitaire. Lorsqu’ils étaient divisés par genre, les détenteurs de DS présentaient des différences importantes. Les résultats de la présente étude appuient les initiatives en cours soulignant la nécessité de respecter l’égalité des genres en chirurgie plastique universitaire.

## Introduction

Diversity is suggested to augment creativity, innovation, problem solving, and performance.^1,2^ Despite this, gender disparity across academic plastic surgery is well documented.^3–5^ Prior research has shown that gender-diverse care teams have higher patient-reported outcome measures.^5^ Given this, it follows that a greater female representation in medicine is quintessential. While the barriers impeding females’ progression on the academic ladder are multifactorial,^6–8^ attaining higher education offers all physicians the opportunity of career advancement, promotions, and increased income.^9^

With the rapidly advancing rate of modern medical care, the average surgeon may choose to add to their credentials and skills. Additional degrees (ADs) have become a popular tool, with many surgeons seeking additional education in order to improve research literacy, leadership competencies, or business skills.^9^ Prior studies have shown that that the presence of an AD is associated with academic career advancement, including greater research productivity, which concurrently allows for more opportunities to climb the academic ladder (eg, promotion to higher professorships) and in turn attain higher compensation.^10^

This advanced education can be particularly beneficial for plastic surgeons.^11^ First, given that plastic surgery is among one of the most competitive and innovative specialties, ADs may allow for a competitive edge over peers.^12,13^ Second, among their many responsibilities, plastic surgeons are tasked with balancing administrative and research endeavors with a high-demanding clinical work.^14^ Often, additional training in business, public health, or research can be an investment for future academic success and potential leadership opportunities.^13^ For women, maternity leave can particularly be an opportunity to pursue additional academic attainment. Third, given the wide encompassing discipline of plastic surgery, it is likely that residents may not be able to explore all parts of the speciality in great detail.^14^ Accordingly, attaining an AD may supplement as an extension of learning for the residency curriculum. Other times, the ADs may be unrelated and provide a focused opportunity to learn in great detail about a new subject.^15^

As the overbearing issue of persistent disparities among plastic surgery faculty still exists,^16^ it is important to question whether the attainment of ADs influences the appointment of medical faculty among Canadian plastic surgery departments. Although prior studies conducted in the United States have shown a rise in advanced degree acquisition among academic plastic surgery faculty,^9^ this has not been documented in the Canadian plastic surgery faculty. The primary objective of this study was to investigate the relationship between attainment of advanced degrees and research productivity, academic ranks, and most notably, gender in Canadian plastic surgery departments.

## Methodology

### Statement of Ethics

This study was exempt from institutional review board approval because all data was extracted from publicly available resources.

### Data Collection

The Canadian Resident Matching Service (CaRMS) and the 2022 Accreditation Council for Graduate Medical Education (ACGME) accredited programs list was searched to compile a list of medical schools that offer plastic surgery training for residency in Canada. From the list of Canadian programs, faculty websites were identified (n = 12) from publicly available program websites and the following information was recorded: faculty name, gender, race, international medical graduate (IMG) status, fellowship status, academic rank, and advanced degree attainment status. Data was also corroborated with publicly available provincial College data to ensure variables such as years in practice are accurately reflected for included faculty. Any missing or outdated information was cross-referenced with online sources such as LinkedIn, Doximity, and Google. Advanced degree attainment was classified as any degree other than a primary medical degree. The data collection period spanned August 2021 to December 2021. Inclusion criteria were academic faculty members with (1) an MD degree or equivalent, (2) plastic surgery training, and (3) are accredited plastic surgeons. Board certification of faculty members was confirmed through a search of the *Canadian Society of Plastic Surgeons* database. Exclusion criteria were programs with websites that lacked accessible faculty listing, or faculty members who were research, voluntary or adjunct faculty, retired or emeritus professors, and instructors who have yet to attain promotion to professorship.

Elsevier's Scopus database was utilized to gather information on faculty members’ total publications, citations, H-index, publication timeline, and years of active research. Scopus was chosen as the database of choice due to its reliability and consistency when compared to Google Scholar or Web of Science.^16,17^ The H-index captures output based on the total number of publications and the total number of citations to those works, providing a focused snapshot of an individual's research performance.

Gender for each faculty member was predicted using Genderize.io API (Application Programming Interface). This validated application predicts the gender of a person through input of their name and location. For the purposes of this study, our probability was set >.85 and required a minimum of 5 counts, similar to previous studies.^7,9^ Names that did not meet these criteria were individually queried on Google, LinkedIn, Doximity, and department-specific websites to determine gender based on gender-specific pronouns. If a gender could not be determined with Genderize.io or from a manual search, they were excluded from further analysis.

### Statistical Analysis

Continuous variables were examined using a 2-tailed Student's *t*-test, and categorical variables were examined using Pearson's chi-square tests. Two-sided *p*-values were used for hypothesis testing, and statistical significance was set at *P* < .05. All statistical analyses were performed using the SPSS software package version 27.0 (SPSS Inc., Chicago, IL).

## Results

**Figure 1.**
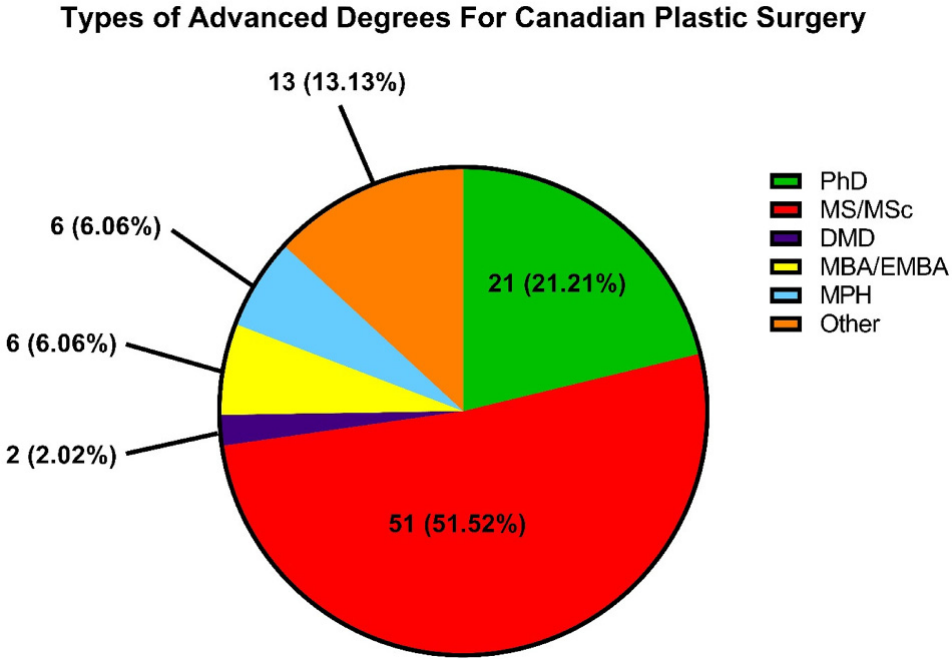
The distribution of advanced degrees among Canadian plastic surgery faculty n (%).

A total of 299 faculty members were identified. Of these, 33% (N = 99) attained an AD (Table 1). From all plastic surgeons, 40% females (N = 38) obtained ADs, while 30% males (N = 61) obtained ADs (*P* = .0402) (Table 1). When controlling for number of years in practice, there was a significantly larger proportion of females than males with ADs as assistant and associate professor (*P* = .0333) (Table 2). Faculty were also compared for academic standing and research productivity. Successful attainment of an AD was associated with a higher number of publications (16 vs 13, *P*-value = .002), a higher H-index (8 vs 3, *P*-value = .002), but similar years of active research (9 vs 9, *P*-value = .811) (Table 3). Among faculty with ADs, most advanced degrees were obtained during (35.0%), or post-residency (38.0%). Fewer ADs were obtained before (20.0%), or during medical school (6.0%) (Table 1). Additionally, assistant professors (40%) and associate professors (47%) had the highest percentage of ADs among ranked faculty (Table 1). The most popular ADs were MSc (52%) and PhDs (21%) (Table 2). The distribution of advanced degrees obtained by plastic surgery faculty is shown in Figure 1. Furthermore, IMGs were less likely than CMGs to not pursue PhDs (*P* = .01). ADs were also most likely to be obtained during or before medical school (*P* = .03) (Table 2). Among degree types, MBAs were obtained more often by males than females (*P* = .05) (Table 2).

**Table 1.** Demographics and Their Corresponding Statistical Significance, n (%).

	No AD	AD	Total	*P-value*
Total	200 (66%)	99 (33%)	299	
Sex				.0402
Male	142 (70%)	61 (30%)	203	
Female	58 (60%)	38 (40%)	96	
International medical graduates				.2268
Yes	186 (68%)	88 (32%)	274	
No	14 (56%)	11 (44%)	25	
MD				.47
Total	200 (67%)	99 (33%)	299	
Advanced degree timing				
Before medical school	N/A	20 (22%)	99	
During medical school	N/A	6 (6%)		
During residency	N/A	35 (35%)		
Post residency	N/A	38 (38%)		
Academic rank				.0024
Assistant professor	70 (60%)	47 (40%)	117	
Associate professor	19 (53%)	17 (47%)	36	
Professor	109 (77%)	32 (23%)	141	
Not found	2 (40%)	3 (60%)	5	

**Table 2.** Research Productivity (*Adjusted for Years in Practice*), Median [IQR].

	With AD	Without AD	*P-value*
Years in practice	11 [4-24]	13 [4-23]	-
Number of publications	16 [4-47]	13 [1-17]	.002
H-index	8 [3-16]	3 [1-9]	.002
Citations	249 [40-789]	64.5 [1-228]	.032

**Table 3.** Demographics of Advanced Degree Types and Their Statistical Significance n (%).

	PhD	MS/MSc	DMD	MBA	MPH	Other
Total	21	51	2	6	6	12
*Sex, P-value*	.67	.90	.26	.05	.15	1.00
Male	12	31	2	6	2	8
Female	9	20	0	0	4	5
*IMG, P-value*	.01	.10	.08	.39	.67	.69
No	15	49	1	6	5	12
Yes	6	2	1	0	1	1
*MD, P-value*	.40	.94	.84	.73	.73	.59
Total	21	51	2	6	6	13
*Timing of degree, P-value*	.25	.26	.03	.59	.55	.54
Before medical school	5 (24%)	10 (20%)	1 (50%)	1 (17%)	0	3 (23%)
During medical school	3 (14%)	1 (2%)	1 (50%)	1 (17%)	0	0
During residency	4 (19%)	24 (47%)	0	1 (17%)	3 (50%)	3 (23%)
Post residency	9 (43%)	16 (31%)	0	3 (50%)	3 (50%)	7 (54%)

## Discussion

This study provides insights into the relationship between attainment of advanced degrees and gender, research productivity, and academic rank. Specifically, there was a significantly larger proportion of females with ADs compared with their male counterparts (*P* = .02). When controlling for number of years in practice, there was a significantly larger proportion of females with ADs as assistant and associate professor than males. This exemplifies that females may be acquiring ADs to have a competitive edge in climbing the academic ladder. This also suggests that other factors besides advanced degrees may be necessary to close the gap between males and females. Consistent with prior studies,^10^ our study revealed higher rank professors are most likely to attain advanced degrees which also indicated that having an AD was significantly associated with having a higher academic rank and greater research productivity.

Academic faculty positions are often coveted positions for various reasons, such as their inherent prestige, opportunities for career advancement, influence on physician training, and the ability to dictate aspects like culture and trajectory of training programs.^18^ There is a growing body of work exploring issues of diversity and inequity by questioning those in positions of influence and, by extension, how this bears out in the literature.^19,20^ Given the rising trend of attaining ADs in plastic surgery over the last 5 years, the role that ADs play in climbing the academic ladder and expanding the functional scope of plastic surgeons has come into question recently.^9,21^ However, publications addressing the Canadian landscape are lacking^5^ and this present study intends to expand on earlier works and advance the conversation to include empirical data.

Current barriers impeding gender parity in academic plastic surgery are complex but may include the limited number of female academic plastic surgeons, which is further complicated by hiring, promotions, and compensation bias.^16^ Our study supports that in order for males and females to hold the same academic rank, a female plastic surgeon may be required to hold an advanced degree. On the other hand, it is also possible that perhaps female surgeons are simply more eager than their counterparts for further education. While acquiring an AD requires a considerable investment of time and effort, it is important to recognize that female surgeons may be inherently disadvantaged due to other responsibilities such as childbearing or home-making.^22^ This study highlights that despite the disproportionate time commitments of female versus male surgeons, more female plastic surgeons have ADs.

While the factors that ultimately drive this thread among female plastic surgeons in Canada are both multifactorial and individual-specific, explanations based on qualitative evidence are likely to provide the best insight at this time. Previous studies have examined a cohort of past and present surgical department chairs.^23^ Their results indicated a theme of internal and external factors that dictate their success in rising to the top tier of academic leadership in their institutions.^23^ Internal factors were identified to include things like self-confidence, adaptability, resilience, and determination and were seen to be instrumental in allowing these women to overcome obstacles they faced when navigating their institutional rise.^24^ Previous work has also noted that women can be more likely to value the perception of “academic citizenship,” particularly in mentor–mentee dyads, than their male counterparts.^24^ This may not only predispose women to seek advanced degrees but also exacerbate the phenomenon seen where female jobseekers are more likely to be dissuaded from applying for positions than their relatively less-qualified male counterparts.^25^ It stands to reason that acquiring an advanced degree could contribute to feeling a firm connection to a scholarly community both institutionally and in a wider sense that would help bolster self-possession and independence for both clinical practice as well as future research pursuits.

External factors also play an understandably large role in the development of leaders within any institution. For women, there remains a significant disparity of expectations when it comes to balancing child-rearing with professional obligations.^26^ Institutional-level policies or attitudes toward childrearing, both explicit and implicit, might significantly influence the likelihood of a woman both pursuing or maintaining places of leadership.^26^ One of the strongest components to success is having access to mentors who can encourage and facilitate upward mobility even as opposition comes from both policies or unspoken gender biases from existing leadership or peers. In absence of outright encouragement and accommodation for realities like maternity leave, the accumulation of microaggressions against women can have a deleterious effect on their achievement without it being explicitly named or upheld by the institution. This can create somewhat of it a nebulous barrier to advancement, which has been seen to require unique and energy-intensive situational workarounds in the professional space.^27^ Finally, it should be noted that in Canada, plastic surgery is among the most competitive specialty to match during the CaRMS (2021) process.^28^ As yet, another hurdle that requires non-specific evaluation of many appropriately qualified applicants, acquiring ADs may play a role in the perceived appeal of applicants when applying to programs, or come as a pursuit that occurs after having not matched in the prior year. The influence of this specialty's competitiveness is speculative only, especially as we lack the data on male–female distinction for timing of AD acquisition and therefore represents an area of meaningful potential future research.

**Figure 2.**
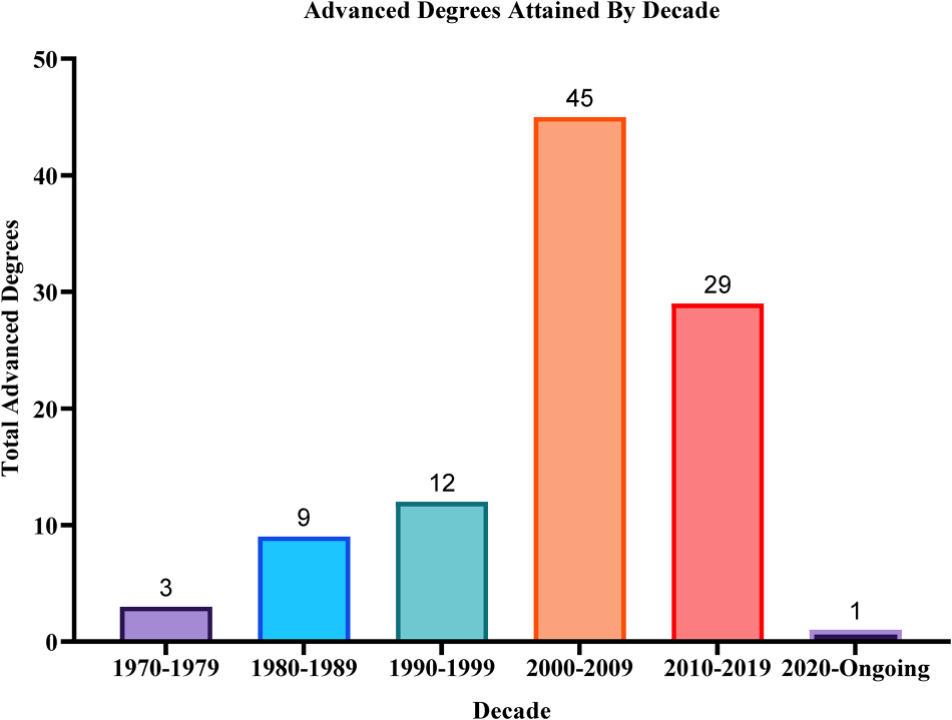
The number of advanced degrees (n), by decade.

Consistent with prior studies research, our study found a significant correlation between ADs and higher number of publications, citations, and H-index (Table 3). This can be expected since an AD is an important notion regarding specific skills for a successful career. For example, having a PhD allows for an in-depth understanding of a specific scientific problem. That knowledge can help facilitate research efforts and funding opportunities.^29^ The most common advanced degreed attained were MSc and PhD, comprising 72% of all ADs attained by Canadian plastic surgeons (Figure 1). While the US plastic surgeons also commonly pursued MSc and PhDs, they were significantly more likely to pursue MBA than Canadian plastic surgeons (*P* = .002). In regard to timing of AD attainment, the majority of ADs attained were either during or after residency (Table 2). In contrast, the US plastic surgeons were likely to pursue PhDs prior to or during medical school. While there may be numerous professional and personal factors driving one's decision to pursue a degree when they did, it is interesting to note that PhD attainment trends stands on opposite ends of the spectrum, between the 2 countries. While obtaining an additional degree (AD) during pre-medical school versus post-residency may be driven by different incentives, future studies may choose to investigate the effect attaining ADs during pre-med have on academic productivity post-residency training. Additionally, it is important to consider that advanced degrees were not common 30 years ago (Figure 2), but likely due to the increased competitiveness to obtain a seat in medical school/residency, and to obtain a job in an academic setting, more trainees may be obtaining advanced degrees.

Whether it be to sharpen one's administrative leadership, entrepreneurial enterprise, or primary clinical research to supplement plastic surgery training, an AD can have plenty to offer plastic surgeons. The latter may be useful for establishing a laboratory or area of focus for one's practice, and the former may tend to contribute less to scholarly activities over the length of a career. The motivation of an individual to pursue an AD is as unique as their circumstance, and therefore its contribution to their practice may be multifocal. Regardless, attaining advanced degrees can be a time consuming, costly, and taxing endeavor for preoccupied plastic surgeons and can therefore be a venture that some surgeons may find disadvantageous. This data may lend itself to speculative explanations, however subsequent qualitative research would best contribute to an understanding of how and why these AD-holders chose to pursue the degree they did. Future research may choose to assess if plastic surgeons who pursued an AD did this to propel their career or if this was incidentally associated with academic institutions. It may also be interesting to investigate the demographic distribution and utility of ADs among community plastic surgeons. A study outlining the timeline of degree completion may also prove value when compared to research productivity before and after degree attainment.

There are several limitations to this study. First, our study used Genderize.io to categorize gender into either male or female with a probability of >.85. Given that gender can be fluid and may change from time to time, we run the risk of our gender data being inaccurate. Systematic errors also include misclassification of authors with a gender neutral first name (eg, Robyn) or names of authors from non-Western country origin (eg, Asia or the Middle East). Second, all data collected is public information and hence, we run the risk of authors’ academic ranking and H-index not being up to date. To ensure accuracy, information was cross-checked on department-specific websites, LinkedIn, and Doximity. Third, assessing longitudinal information on advanced academic degrees can be difficult, given that faculty members can be working to obtain a degree at any time. Some faculty may have retired and be removed from faculty websites and therefore, be missed in our data pool. We are limited by the validity of information published by program websites, their recency, or their online existence altogether. Lastly, the exemption of adjunct faculty served to focus analysis on full-time faculty among healthcare institutions. However, adjunct faculty are rapidly being relied upon within modern academia with an approximate increase by over 100% representation over the last 3 decades.^30^ Combined with the varying methods of ranking, promotion, and time allocation constructed by individual institutions, this significant segment of faculty could potentially represent a skew in the trends seen within the data. Despite these limitations, understanding problems of equity within complex systems by beginning with a simplistic approach is often the most realistic way to initiate conversations and generate new questions that may ultimately produce meaningful change.^6^

## Conclusion

Our study suggests about one-third of Canadian plastic surgeons have ADs. We also note that ADs can be helpful for scholarly productivity and may contribute to holding coveted leadership positions within their institutions. Despite controlling for number of years in practice and academic rank, females are more likely than males to have advanced degrees. The gender disparity continues to be perpetuated and highlights the importance of equity, diversity, and inclusion within the systems that train plastic surgeons. The results of this study will lend support to ongoing endeavors voicing the need of gender equity in academic plastic surgery.
